# Does the presence of tumor-induced cortical bone destruction at CT have any prognostic value in newly diagnosed diffuse large B-cell lymphoma?

**DOI:** 10.1007/s00256-015-2102-z

**Published:** 2015-02-07

**Authors:** Hugo J. A. Adams, John M. H. de Klerk, Rob Fijnheer, Ben G. F. Heggelman, Stefan V. Dubois, Rutger A. J. Nievelstein, Thomas C. Kwee

**Affiliations:** 1 Department of Radiology and Nuclear Medicine, University Medical Center Utrecht, Heidelberglaan 100, 3584 CX Utrecht, The Netherlands; 2 Department of Nuclear Medicine, Meander Medical Center, Amersfoort, The Netherlands; 3 Department of Hematology, Meander Medical Center, Amersfoort, The Netherlands; 4 Department of Radiology, Meander Medical Center, Amersfoort, The Netherlands; 5 Department of Pathology, Meander Medical Center, Amersfoort, The Netherlands

**Keywords:** Bone marrow, Cortical bone, CT, Diffuse large B-cell lymphoma, Prognosis

## Abstract

**Purpose:**

To determine the prognostic value of tumor-induced cortical bone destruction at computed tomography (CT) in newly diagnosed diffuse large B-cell lymphoma (DLBCL).

**Materials and methods:**

This retrospective study included 105 patients with newly diagnosed DLBCL who had undergone CT and bone marrow biopsy (BMB) before R-CHOP (rituximab, cyclophosphamide, hydroxydaunorubicin, Oncovin, and prednisolone) chemo-immunotherapy. Cox regression analyses were used to determine the associations of cortical bone status at CT (absence vs. presence of tumor-induced cortical bone destruction), BMB findings (negative vs. positive for lymphomatous involvement), and dichotomized National Comprehensive Cancer Network International Prognostic Index (NCCN-IPI) strata (low risk vs. high risk) with progression-free survival (PFS) and overall survival (OS).

**Results:**

Univariate Cox regression analysis indicated that cortical bone status at CT was no significant predictor of either PFS or OS (*p* = 0.358 and *p* = 0.560, respectively), whereas BMB findings (*p* = 0.002 and *p* = 0.013, respectively) and dichotomized NCCN-IPI risk strata (*p* = 0.002 and *p* = 0.003, respectively) were significant predictors of both PFS and OS. In the multivariate Cox proportional hazards model, only the dichotomized NCCN-IPI score was an independent predictive factor of PFS and OS (*p* = 0.004 and *p* = 0.003, respectively).

**Conclusions:**

The presence of tumor-induced cortical bone destruction at CT was not found to have any prognostic implications in newly diagnosed DLBCL.

## Introduction

Diffuse large B-cell lymphoma (DLBCL) is the most common non-Hodgkin lymphoma, making up approximately 30 to 35 % of all cases [1, 2]. Diagnosis of bone marrow involvement is important in DLBCL, because it is an adverse prognostic factor with potential consequences for treatment planning [1–3]. Blind bone marrow biopsy (BMB) of the posterior iliac crest is therefore a standard staging procedure in these patients [4]. ^18^ F-fluoro-2-deoxy-D-glucose positron emission tomography/computed tomography (FDG-PET/CT) may be used complementary or as a partial alternative to BMB for the detection of bone marrow evaluation in DLBCL [5]. Unfortunately, several recent studies have indicated that, unlike BMB-based bone marrow status, FDG-PET/CT-based bone marrow status is not predictive of progression-free survival (PFS) and overall survival (OS) [6–8]. Although the reason for the lack of prognostic value of FDG-PET/CT in this setting is still unclear, it indicates that focal FDG-avid bone marrow lesions that are presumed to be lymphomatous are clinically irrelevant in this context.

CT, either alone or in combination with FDG-PET, is a compulsory staging procedure in DLBCL [4]. Although CT is not an appropriate method to assess the bone marrow and to detect lymphomatous lesions that are confined to the bone marrow [9], it is the best method to evaluate the cortical bone. CT scans of patients with DLBCL sometimes show tumor-induced cortical bone destruction, either as a result of lymphoma extending from the bone marrow to the bony cortex, or as a result of extramedullary lymphoma growing into the adjacent bony cortex. Although cortical destruction shown at CT indicates that the underlying DLBCL has an aggressive tumor growth, the prognostic implications of this finding are still unclear.

The purpose of this study was to determine the prognostic value of tumor-induced cortical bone destruction at CT in newly diagnosed DLBCL patients who are treated with R-CHOP (rituximab, cyclophosphamide, hydroxydaunorubicin, Oncovin, and prednisolone) chemo-immunotherapy.

## Materials and methods

### Study design and patient population

This retrospective study was approved by the local institutional review board and the requirement for written informed consent was waived. All patients with newly diagnosed DLBCL routinely undergo pretreatment CT at our institution. The hospital’s database (which identifies patients on the basis of their clinical diagnosis) was searched for all patients who were newly diagnosed with DLBCL between January 2004 and December 2013. Study inclusion criteria were: newly diagnosed and histologically proven DLBCL, availability of CT (either full-dose CT or low-dose CT as part of an FDG-PET examination) from skull base to upper thigh, availability of blind bone marrow biopsy (BMB) of the iliac crest with a time interval between CT and BMB <30 days, availability of serum lactate dehydrogenase (LDH) measurement, and treatment with the R-CHOP regimen. Study exclusion criteria were: primary mediastinal DLBCL (which is regarded as a different disease entity), previously treated/relapsed lymphoma, transformed lymphoma, coexistence of another lymphoma subtype in the diagnostic biopsy, another malignancy within the previous 5 years, and start of treatment before CT.

### CT acquisition

CT scans were acquired using either a stand-alone 16-detector row CT system (Somatom Sensation 16, Siemens Healthcare, Erlangen, Germany) or a 40-detector row integrated PET/CT system (Biograph 40 TruePoint PET/CT, Siemens Healthcare, Erlangen, Germany). All patients ingested 780 ml of oral contrast agent (750 ml of water mixed with 30 ml of Telebrix Gastro, Guerbet, Gorinchem, The Netherlands), 2 h before image acquisition. Full-dose CT from skull base to upper thigh was performed in the portal venous phase, following intravenous administration of a non-ionic iodinated contrast agent (Omnipaque 300 [GE Healthcare, Eindhoven, the Netherlands] for Somatom Sensation 16 or Xenetix 300, [Guerbet, Gorinchem, The Netherlands] for Biograph 40 TruePoint PET/CT), using the following settings: 120 kV, 90 mA (Somatom Sensation 16) or 60–160 mAs (automatic dose modulation) (Biograph 40 TruePoint PET/CT), pitch of 0.8, 0.5-s (Somatom Sensation 16) or 0.8-s (Biograph 40 TruePoint PET/CT) tube rotation time, and 1.5-mm slice width. Typical CTDI_vol_ values for full-dose CT scanning was around 4.0 mGy for premonitoring of the intravenous bolus, 28.3 mGy for monitoring of the intravenous bolus, 6.5 mGy for neck CT, 7.6 mGy for chest CT, and 13.3 mGy for abdomen CT. A subset of patients only underwent non-intravenous contrast-enhanced low-dose CT as part of their FDG-PET/CT examination, which was acquired with the following settings: 120 kV, 26–30 mAs (automatic dose modulation), pitch of 1.2, 0.8-s tube rotation time, and 1.5-mm slice width. Typical CTDI_vol_ value for low-dose CT scanning was around 1.7 mGy. A smooth reconstruction kernel was used, and CT images were reconstructed to contiguous axial, coronal, and sagittal 5-mm slices.

### Image interpretation

**Fig. 1:**
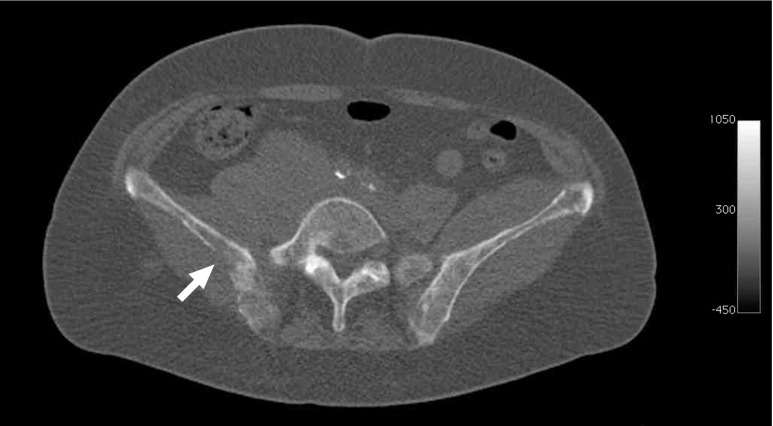
Example of tumor-induced cortical destruction at CT in a 69-year-old woman with newly diagnosed DLBCL. Axial CT shows pathological densities in both iliac wings, indicating lymphomatous bone marrow involvement, and a focal disruption of the bony cortex (*arrow*)

**Fig. 2:**
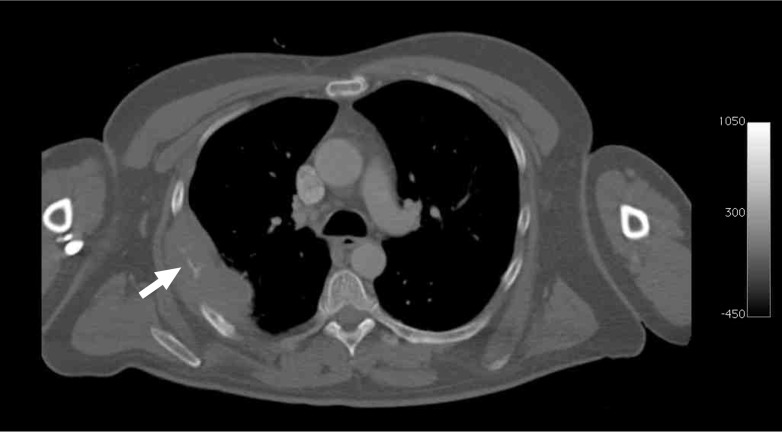
Example of tumor-induced cortical destruction at CT in a 49-year-old man with newly diagnosed DLBCL. Axial CT shows focal destruction of the right fifth rib (*arrow*) with surrounding soft tissue mass

A reader with more than 5 years of clinical CT experience (T.C.K) evaluated the CT images for the presence of tumor-induced cortical bone destruction. The reader was blinded to clinical, laboratory, and other imaging findings, and patient outcome. Tumor-induced cortical destruction was defined as non-integrity of the bony cortex with underlying hypodense (lytic) bone marrow changes, with or without associated tumor mass in the surrounding soft tissues (Figs. 1 and 2). Vertebral body collapse was only considered positive for tumor-induced cortical destruction if focal or epidural soft tissue masses and/or involvement of the pedicles was also present [10]. If present, the maximum diameter in any plane of the largest area of cortical bone destruction was measured. The reader also evaluated all CT scans for the presence and extent of nodal and extramedullary extranodal disease. For this purpose, any lymph node measuring more than 10 mm in short-axis diameter in the axial plane was considered positive for lymphoma [11]. Focal areas with abnormal attenuation and masses in extranodal organs were also considered positive for lymphoma, excluding obviously benign lesions such as cysts and hemangiomas. Splenomegaly (splenic index exceeding 725 cm^3^ [12]) was regarded positive for lymphoma, but hepatomegaly was not. Subsequently, presence of extranodal disease in major organs (bone marrow, central nervous system, liver/gastrointestinal tract or lung) and Ann Arbor stage (stage I-IV) [13] were recorded for subsequent National Comprehensive Cancer Network (NCCN) International Prognostic Index (IPI) scoring [3].

### BMB

Unilateral BMB of the posterior iliac crest was acquired by different hematologists and evaluated by different hematopathologists, as part of standard care.

### NCCN-IPI

Age, serum LDH levels, presence of extranodal disease in major organs (either bone marrow, central nervous system, liver/gastrointestinal tract or lung involvement), presence of Ann Arbor stage III/IV disease, and Eastern Cooperative Oncology Group (ECOG) performance status score of each patient were recorded at time of diagnosis to calculate an NCCN-IPI score [3]. For the purpose of this study, only histologically confirmed bone marrow involvement, and not imaging-based bone marrow involvement, was used to define stage IV disease and extranodal involvement, as recommended by Zhou et al. [3]. The four NCCN-IPI risk groups (low risk [scores 0–1], low-intermediate risk [scores 2–3], high-intermediate risk [scores 4–5] and high risk [scores 6–8]) were dichotomized into low risk (comprising low and low-intermediate risk patients) and high risk (comprising high-intermediate and high-risk patients) groups.

### Patient follow-up

Clinical follow-up and either follow-up stand-alone FDG-PET with stand-alone CT or integrated FDG-PET/CT were used in all patients to determine if and when relapsed or progressive disease had occurred, in line with the Revised Response Criteria for Malignant Lymphoma [14]. PFS was calculated from the date of diagnosis to documented disease relapse/progression or, for patients dying as a result of causes unrelated to DLBCL or the lymphoma treatment, the date of death. For surviving patients who did not experience disease relapse/progression, follow-up was censored at the date the patient was last known to be alive. OS was calculated from the date of diagnosis until death as a result of any cause or, in surviving patients, censored at the date last known to be alive.

### Statistical analysis

Associations between cortical bone status at CT (absence vs. presence of tumor-induced cortical bone destruction) and the NCCN-IPI factors categorized age (≤40, 40–60, 60–75, and >75 years), categorized LDH ratio (≤1, 1–3 or >3 upper limit of normal), presence of extranodal disease in major organs (either bone marrow [histologically confirmed], central nervous system, liver/gastrointestinal tract or lung), presence of Ann Arbor stage III/IV disease, and ECOG performance status (≥2) were assessed using Spearman (ρ) (for ordinal NCCN-IPI factors) or Phi (φ) (for binary NCCN-IPI factors) correlation coefficient analyses [15].

PFS and OS were analyzed using the Kaplan–Meier method with log-rank test for comparison of differences [16], according to cortical bone status at CT (absence vs. presence of tumor-induced cortical bone destruction), BMB findings (negative vs. positive for lymphomatous involvement), and dichotomized NCCN-IPI risk strata (low risk vs. high risk). Subsequently, univariate and multivariate Cox regression analyses were performed to determine the influence of cortical bone status at CT, BMB findings, and dichotomized NCCN-IPI risk strata on PFS and OS.

Two-sided *p* values less than 0.05 were considered to indicate a statistically significant difference. Statistical analyses were executed using MedCalc statistical software version 12.6.0 (Ostend, Belgium).

## Results

### Patients

A total of 208 patients were newly diagnosed with DLBCL between January 2004 and December 2013. Of these 208 patients, four were excluded because of primary mediastinal DLBCL, 15 were excluded because of transformed lymphoma, 18 were excluded because of coexistence of another lymphoma subtype in the diagnostic biopsy, four were excluded because of another malignancy within the previous 5 years, 23 were excluded because of non-availability of CT from skull base to upper thigh, 22 were excluded because of lack of BMB, four were excluded because of a BMB of poor, non-diagnostic quality, three were excluded because the time interval between CT and BMB exceeded 30 days, and ten were excluded because they received no or another treatment than R-CHOP regimen. Finally, 105 patients (57 men and 48 women, mean age, 63.9 years, age range, 24–87 years) were included. Detailed patient characteristics are shown in Table 1.

**Table 1:** Characteristics of included patients

	Tumor-induced cortical destruction at CT	No tumor-induced cortical destruction at CT
Patients (no.)	8	97
Age		
Mean ± SD (years)	68.4 ± 12.0	63.6 ± 13.9
Median (years)	68.5	65.0
Range (years)	49–83	24–87
Male/Female	3/5	54/43
Positive/negative BMB	3/5	16/81
NCCN-IPI factors		
Age		
≤40 years	0	7
>40 to ≤60 years	2	27
>60 to ≤75 years	3	44
>75 years	3	19
ECOG PS > 1	1	16
LDH > ULN		
≤1	4	28
>1 to ≤3	4	56
>3	0	13
Stage III/IV	7	78
Major extranodal involvement**	6	46
NCCN-IPI score		
Low risk (0–1)	0	8
Low-intermediate risk (2–3)	2	22
High-intermediate risk (4–5)	4	49
High risk (≥6)	2	18
Follow-up time of surviving patients		
Mean ± SD (days)	647 ± 490	1,542 ± 914
Median (days)	511	1325
Range (days)	313–1,679	134–3,597
Disease relapse or progression, or death (No.)	1	36
Death (No.)	1	32

*For the purpose of this study, only histologically proven bone marrow involvement was used to define stage IV disease, as recommended by Zhou et al. [3]. Imaging-based bone marrow involvement only (i.e., the presence of tumor-induced cortical bone destruction at CT) was not used to define stage IV disease

**Defined as either bone marrow, central nervous system, liver/gastrointestinal tract or lung involvement, with histological confirmation of bone marrow and central nervous system involvement, as recommended by Zhou et al. [3]

*BMB* bone marrow biopsy, *ECOG PS* Eastern Cooperative Oncology Group Performance Score, *LDH* lactate dehydrogenase, *NCCN-IPI* National Comprehensive Cancer Network International Prognostic Index, *SD* standard deviation, *ULN* upper limit of normal (according to the local reference values of each participating institution)

### Cortical bone status at CT

Tumor-induced cortical bone destruction at CT was present in eight of 105 (7.6 %) patients, and was located in the cranium (*n* = 1), nasal bone (*n* = 1), mandible (*n* = 1), clavicle (*n* = 1), ribs (*n* = 1), iliac wings (*n* = 1) and sacrum (*n* = 2). An associated tumor mass in the surrounding soft tissues was found in three of these eight (37.5 %) patients. The mean maximum diameter of the largest area of cortical bone destruction in those eight patients was 3.5 cm (range, 2–4.5 cm). Representative examples are shown in Figs. 1 and 2.

### BMB

BMB was positive for lymphoma in 19 of 105 (18.1 %) patients. Only 3/19 (16 %) patients had tumor-induced cortical bone destruction.

### Correlations between cortical bone status at CT and NCCN-IPI factors

There were no correlations between cortical bone status at CT and the NCCN-IPI factors categorized age (ρ = 0.111, *p* = 0.261) categorized LDH ratio (ρ = −0.146, *p* = 0.138), presence of extranodal disease in major organs (φ = 0.146, *p* = 0.136), presence of Ann Arbor stage III/IV disease (φ = 0.048, *p* = 0.625), and ECOG performance status (φ = −0.029, *p* = 0.769).

### Patient follow-up

The median follow-up time of surviving patients was 1,454 days (range, 134–3,597 days). Of 105 patients, 37 (35.2 %) experienced disease relapse/death, and 33 patients (31.4 %) died (Table 1).

### Prognostic value of tumor-induced cortical bone destruction at CT

**Fig. 3:**
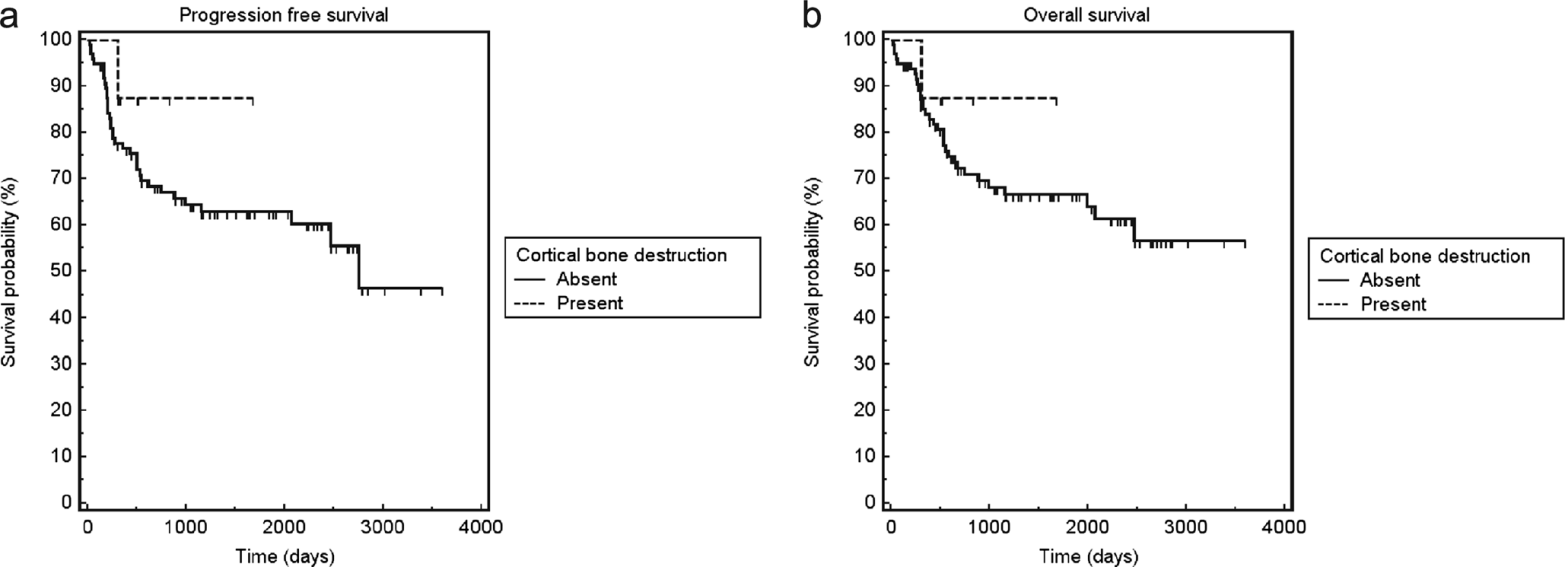
Kaplan–Meier curves for PFS (**a**) and OS (**b**) of patients with tumor-induced cortical destruction at CT vs. patients without tumor-induced cortical destruction at CT. Patients with tumor-induced cortical destruction at CT had no significantly different PFS and OS than those without (log-rank test, *p* = 0.341 and *p* = 0.554, respectively)

**Fig. 4:**
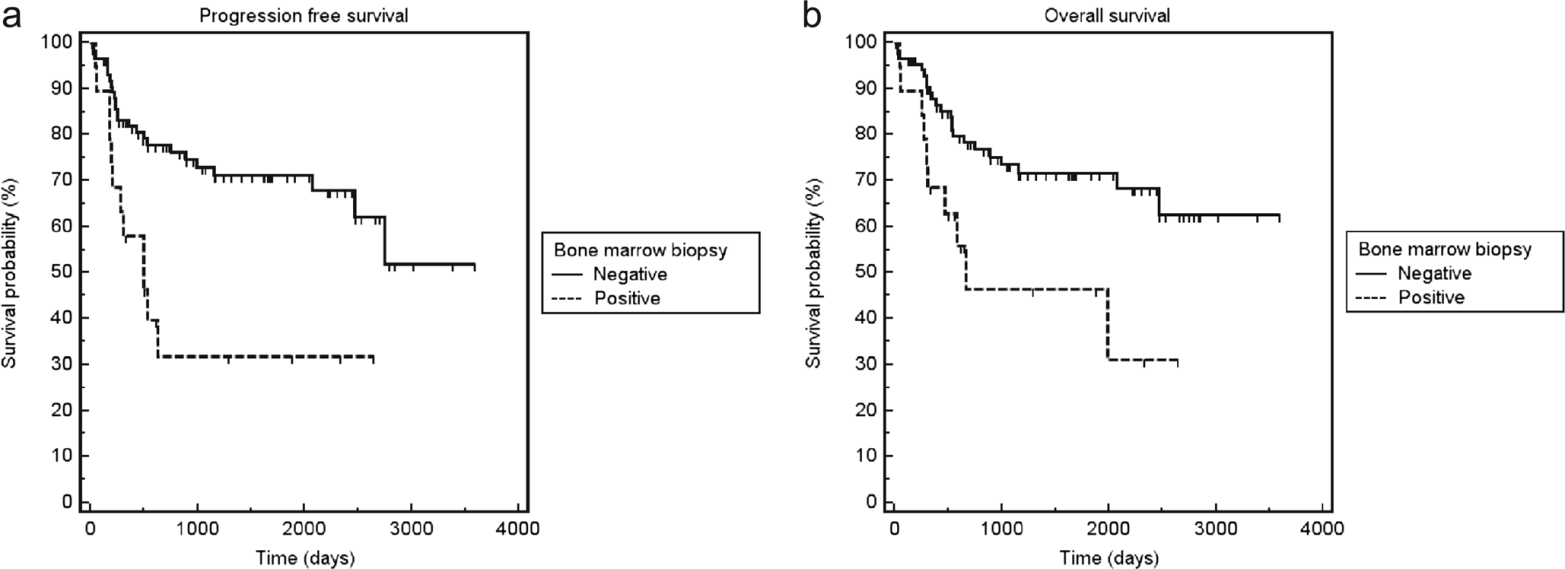
Kaplan–Meier curves for PFS (**a**) and OS (**b**) of patients with negative BMB vs. those with positive BMB. Patients with a positive BMB had significantly worse PFS and OS than those with a negative BMB (log-rank test, *p* = 0.001 and *p* = 0.001, respectively)

**Fig. 5:**
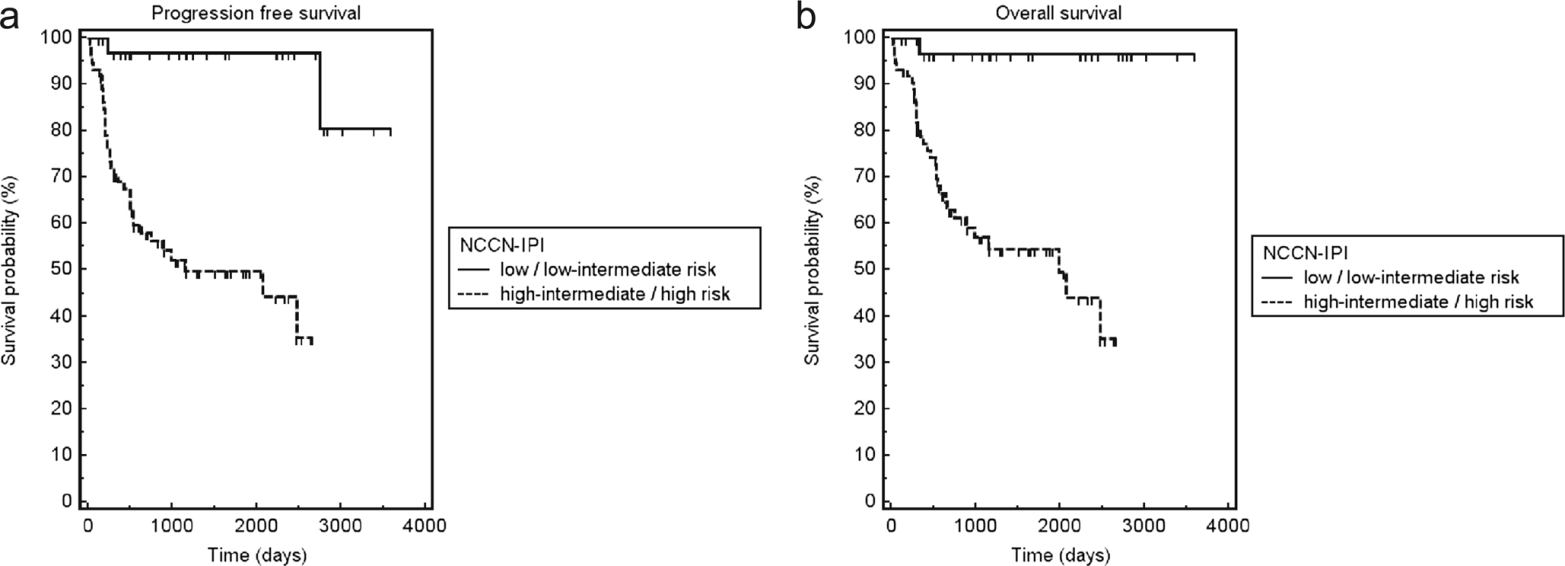
Kaplan–Meier curves for PFS (**a**) and OS (**b**) of patients with low risk NCCN-IPI scores vs. high-risk NCCN-IPI scores. Patients with high-risk NCCN-IPI scores had significantly worse PFS and OS than those with low risk NCCN-IPI scores (log-rank test, *p* < 0.001 and *p* < 0.001, respectively)

Patients with tumor-induced cortical bone destruction at CT had no significantly different PFS and OS than those without (log-rank test, *p* = 0.341 and *p* = 0.554, respectively) (Fig. 3). PFS and OS were significantly worse in patients with a positive BMB (log-rank test, *p* = 0.001 and *p* = 0.001) (Fig. 4) and in patients with a high-risk NCCN-IPI score (log-rank test, *p* < 0.001 and *p* < 0.001, respectively) (Fig. 5). Univariate Cox regression analysis indicated that cortical bone status at CT was no significant predictor of either PFS or OS (*p* = 0.358 and *p* = 0.560, respectively), whereas BMB findings (*p* = 0.002 and *p* = 0.013, respectively) and dichotomized NCCN-IPI risk strata (*p* = 0.002 and *p* = 0.003, respectively) were significant predictors of both PFS and OS (Table 2). In the multivariate Cox proportional hazards model, only the dichotomized NCCN-IPI score was an independent predictive factor of PFS and OS (*p* = 0.004 and *p* = 0.003, respectively) (Table 3).

**Table 2:** Cox regression analysis of PFS

Characteristic	Univariate analysis			Multivariate analysis		
	Hazard ratio	95 % CI	*p* value	Hazard ratio	95 % CI	*p* value
Tumor-induced cortical destruction at CT vs. no tumor-induced cortical destruction at CT	0.393	0.054–2.854	0.358	–	–	–
BMB positive vs. BMB negative	3.013	1.501–6.048	0.002	–	–	–
NCCN-IPI high risk vs. NCCN-IPI low risk	21.774	3.000–158.078	0.002	21.774	3.000–158.078	0.002

**Table 3:** Cox regression analysis of OS

Characteristic	Univariate analysis			Multivariate analysis		
	Hazard ratio	95 % CI	*p* value	Hazard ratio	95 % CI	*p* value
Tumor-induced cortical destruction at CT vs. no tumor-induced cortical destruction at CT	0.551	0.075–4.033	0.559	–	–	–
BMB positive vs. BMB negative	2.588	1.229–5.448	0.013	–	–	–
NCCN-IPI high risk vs. NCCN-IPI low risk	20.186	2.768–147.191	0.003	20.186	2.768–147.191	0.003

## Discussion

The results of this study show that the incidence of tumor-induced cortical bone destruction is low (i.e., less than 10 %) in newly diagnosed DLBCL. Interestingly, cortical bone destruction was observed in only 16 % (three out of 19 patients) with BMB-proven bone marrow involvement. Thus, in most patients, bone marrow involvement does not result in osteolysis. Most importantly, tumor-induced cortical bone destruction is not associated with any of the established NCCN-IPI factors, and is not associated with a worse PFS or OS. It can be speculated that although cortical bone destruction indicates an aggressive tumor growth, it does not indicate the presence of an R-CHOP-resistant phenotype. On the other hand, both BMB findings and the (dichotomized) NCCN-IPI risk strata were associated with PFS and OS, with BMB-positive patients and patients with high-risk NCCN-IPI scores showing worse outcome than BMB-negative patients and patients with low-risk NCCN-IPI scores, respectively. However, unlike BMB status, only the NCCN-IPI risk score remained as an independent predictive factor of both PFS and OS, which is in line with the recent publication by Zhou et al. [3]. The latter finding can be explained by the fact that BMB status is already part of the NCCN-IPI [3].

Given the fact that approximately one-third of patients with DLBCL still develop relapsed/refractory disease [1, 2], efforts are being made to identify new prognostic biomarkers that improve risk stratification in this disease. Unlike imaging-based bone marrow involvement, the presence of histologically confirmed bone marrow involvement is already an established negative prognostic factor in DLBCL, and is part of the current NCCN-IPI [3]. A disadvantage of BMB, however, is the fact that it assesses only a very small part of the bone marrow, thus being prone to sampling errors. Cross-sectional whole-body imaging modalities like FDG-PET and magnetic resonance imaging (MRI) have the advantage of being able to visualize the entire bone marrow, and it was hoped that bone marrow assessment with these techniques would improve prognostication. Although the role of MRI in this setting needs further investigation [17], the majority of studies on this topic have indicated that FDG-PET/CT-based bone assessment has no prognostic implications in DLBCL [6–8, 18]. CT is not an appropriate technique to evaluate the bone marrow [9], but is an ideal method to evaluate the bony cortex. Although lymphoma-induced cortical bone destruction indicates aggressive tumor growth, the hypothesis that this would be associated with a poorer outcome was not supported by the findings of the present study.

The prognostic impact of cortical bone destruction has been examined previously by Lee et al. [19]. Their study included patients diagnosed with DLBCL between 1994 and 2009. The PFS of 60 patients with bone lesions was compared with those of 181 patients with stage IV disease and similar IPI scores but without bone destruction. The PFS was not significantly different (*p* = 0.457) between two groups. Lee et al. [19] concluded that bone involvement may not have an additive adverse impact on the prognosis of Ann Arbor stage IV DLBCL, both in their entire study population (which included patients treated with different therapies) as well as in the subgroup of patients treated with R-CHOP. However, drawbacks of the study by Lee et al. [19] are that criteria for bone involvement were not reported, that the prognostic impact of bone involvement was assessed using matched controls rather than assessing the additional impact in the entire group of DLBCL patients over de NCCN-IPI and other risk factors using Cox regression, and that controls were selected on the basis of the old IPI [20] rather than the recently published NCCN-IPI [3].

This study had several limitations. First, the number of patients with cortical bone destruction was low. A future study with sufficient power may be necessary to confirm the present findings. Second, although the majority of patients had undergone full-dose CT, several patients received a low-dose CT scan only, as part of their FDG-PET/CT examination. In addition, a smooth reconstruction kernel was used and CT images were reconstructed to 5-mm slices. These factors may have had a negative impact on bony lesion detection. Third, although the NCCN-IPI defines four risk groups (low risk, low-intermediate risk, high-intermediate risk, and high risk), patients were dichotomized in low-risk (including low and low-intermediate risk patients) and high-risk (including high-intermediate and high risk patients) groups for survival analyses, because of the relatively limited sample size of the present study.

In conclusion, the presence of tumor-induced cortical bone destruction at CT was not found to have any no prognostic implications in newly diagnosed DLBCL. The NCCN-IPI remains the most important method for the prognostication of this disease.
